# Marine Compound Catunaregin Inhibits Angiogenesis through the Modulation of Phosphorylation of Akt and eNOS *in vivo* and *in vitro*

**DOI:** 10.3390/md12052790

**Published:** 2014-05-12

**Authors:** Jun-Xiu Liu, Min-Qi Luo, Meng Xia, Qi Wu, Si-Mei Long, Yaohua Hu, Guang-Chun Gao, Xiao-Li Yao, Mian He, Huanxing Su, Xiong-Ming Luo, Shu-Zhong Yao

**Affiliations:** 1 Department of Obstetrics and Gynecology, The First Affiliated Hospital, Sun Yat-sen University, Guangzhou 510080, China; E-Mails: liujxiu@mail.sysu.edu.cn (J.-X.L.); xiameng25@163.com (M.X.); gz87335487@163.net (M.H.); 2 Department of Clinical Laboratory, The Third Affiliated Hospital, Sun Yat-sen University, Guangzhou 510630, China; E-Mail: zhanglmq@sina.com; 3 Guangdong Key Laboratory for Diagnosis and Treatment of Major Neurological Diseases, Department of Neurology, National Key Clinical Department and Key Discipline of Neurology, The First Affiliated Hospital, Sun Yat-sen University, Guangzhou 510080, China; E-Mails: wuqi@medmail.com.cn (Q.W.); ivylsm@163.com (S.-M.L.); liliyao71@163.com (X.-L.Y.); 4 State Key Laboratory of Quality Research in Chinese Medicine, Institute of Chinese Medical Sciences, University of Macau, Macao 999078, China; E-Mails: yaohuahu225@aliyun.com (Y.H.); Huanxingsu@umac.mo (H.S.); 5 Jiaxing University College of Medicine, Jiaxing 314001, China; E-Mail: gaogcjx@163.com; 6 CAS Key Laboratory of Tropical Marine Bio-resources and Ecology, South China Sea Institute of Oceanology, Chinese Academy of Sciences, Guangzhou 510301, China

**Keywords:** anti-angiogenesis, catunaregin, VEGF, zebrafish, HUVECs

## Abstract

Angiogenesis is the formation of blood vessels from pre-existing vasculature. Excessive or uncontrolled angiogenesis is a major contributor to many pathological conditions whereas inhibition of aberrant angiogenesis is beneficial to patients with pathological angiogenesis. Catunaregin is a core of novel marine compound isolated from mangrove associate. The potential anti-angiogenesis of catunaregin was investigated in human umbilical vein endothelial cells (HUVECs) and zebrafish. HUVECs were treated with different concentrations of catunaregin in the presence or absence of VEGF. The angiogenic phenotypes including cell invasion cell migration and tube formation were evaluated following catunaregin treatment in HUVECs. The possible involvement of AKT, eNOS and ERK1/2 in catunaregin-induced anti-angiogenesis was explored using Western blotting. The anti-angiogenesis of catunaregin was further tested in the zebrafish embryo neovascularization and caudal fin regeneration assays. We found that catunaregin dose-dependently inhibited angiogenesis in both HUVECs and zebrafish embryo neovascularization and zebrafish caudal fin regeneration assays. In addition, catunaregin significantly decreased the phosphorylation of Akt and eNOS, but not the phosphorylation of ERK1/2. The present work demonstrates that catunaregin exerts the anti-angiogenic activity at least in part through the regulation of the Akt and eNOS signaling pathways.

## 1. Introduction

Angiogenesis is the formation of blood vessels from pre-existing vasculature. The process of angiogenesis is very complex and tightly controlled under physiological conditions. Excessive or uncontrolled angiogenesis is associated with various pathological conditions including cancer, diabetic retinopathy, and rheumatoid arthritis [1]. Thus, development of novel anti-angiogenesis could greatly benefit human health. For example, cancer is one of the most devastating diseases, and can be a great burden on modern society, while anti-angiogenic therapy is becoming a powerful treatment approach in cancer therapy.

Traditionally, great efforts have been placed on developing drugs that attack and kill cancer cells. During past years, chemotherapy based on cytotoxic agents has been the main treatment for most cancer patients. Although cytotoxic agents have significantly improved cancer survival, these conventional anti-cancer drugs often cause serious side effects, chemo- and radio-resistance, disease relapse and metastases. Therefore, the alternative chemotherapeutic regimens with minimal side effects are currently the priority consideration for the development of cancer treatment. Angiogenesis plays a critical role in the progression of cancer. Through stimulating blood vessel growth from nearby pre-existing capillaries, tumors achieve sufficient blood supply for cancer cell progression and metastasis [2]. It has been shown that increased angiogenesis is associated with poor prognosis and relapse of cancer whereas blocking the formation of new blood vessels prevents the growth of cancer. Thus, the development of inhibitors of angiogenesis is increasingly receiving attention in the field of cancer. In the U.S., there are currently thirteen approved anti-angiogenesis therapies in oncology. However, as current anti-angiogenesis therapy is still in the infant stage of the drug development, there are lots of limitations of anti-angiogenesis therapy such as low efficacy, and development of resistance during the long term use of these therapeutic agents. Therefore, intensive efforts are needed to develop novel anti-angiogenic agents, especially small molecule targeting the tumor vasculature [3].

Vascular endothelial growth factor (VEGF) is a very important stimulator for tumor angiogenesis. VEGF is regarded as the prognostic indicator in cancers because of its significant association with tumor size and progress in several cancers. Thus, VEGF signaling pathway is an attractive target for the development of anti-angiogenesis inhibitors.

The marine environment is a rich source of novel and unusual secondary metabolites for drug discovery. Catunaregin was first isolated from the stem bark of *catunaregam spinosa*, a Chinese mangrove associate and the extraction and isolation of catunaregin was described in the literature [4]. Our previous study demonstrated that catunaregin may have anti-cancer property. In this study, we investigated the potential inhibitory action of catunaregin on angiogenesis in human umbilical vein endothelial cells (HUVECs) and transgenic zebrafish (Danio rerio; fli1:EGFP). We further explored the mechanism by which catunaregin inhibited angiogenesis in HUVECs. We found that catunaregin exerted anti-angiogenic activity in both HUVECs and zebrafish. In addition, catunaregin significantly reduced the phosphorylation of Akt and eNOS and the inhibition was in parallel to its anti-angiogenic effect, suggesting that catunaregin inhibited angiogenesis possibly through the modulation of the Akt and eNOS signaling pathways. Since angiogenesis plays an important pathological role in the progress of a wide range of diseases, our findings provide a rationale for future development of this compound as a potential drug or chemopreventive supplement to target diseases with excessive angiogenesis.

## 2. Results and Discussion

### 2.1. The Isolation and Preparation of Catunaregin

**Chart 1:**
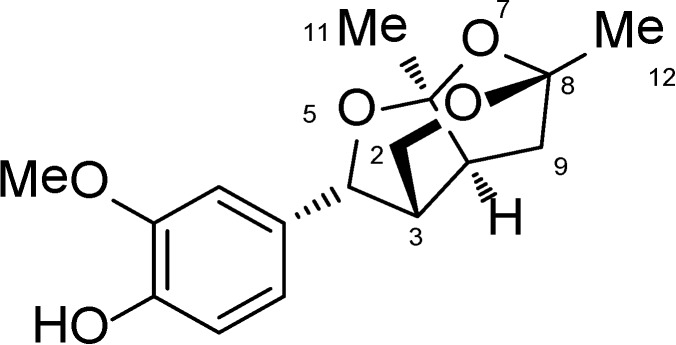
Chemical structure of catunaregin.

Catunaregin was first isolated from the stem bark of *Catunaregam spinosa*, a Chinese mangrove associate and the extraction and isolation of catunaregin was described in the experimental part of the literature [4]. In continuation of our studies on the chemical diversity of mangrove plants in Hainan Island, the plant *Micromelum falcatum* (Lour.) Tan was investigated, and the novel norneolignan, catunaregin was isolated again from this plant. The air-dried material *Micromelum falcatum* (Lour.) Tan (10.0 kg), which was collected in Wenchang, Hainan Province, was extracted with 95% EtOH three times. The aq. residue was subjected to extraction with *n*-hexane and EtOAc (each 3×). The EtOAc extract (102 g) was separated on silica gel (1300 g, 200–300 mesh) with solvents of increasing polarity: 10%–70% acetone in n-hexane and gained 20 fractions. Fr.3 (1.1 g, eluted with *n*-hexane–acetone 7:3) was fractionated on silica gel with chloroform–acetone (19:1), then purified by Semi-preparation HPLC (250 × 10 mm i.d. 5 μm, MeOH/H_2_O, 60:40) to 12.0 mg of catunaregin (Chart 1).

### 2.2. Inhibitory Effect of Catunaregin on VEGF-induced HUVEC Invasion, Migration, and Tube Formation

**Figure 1:**
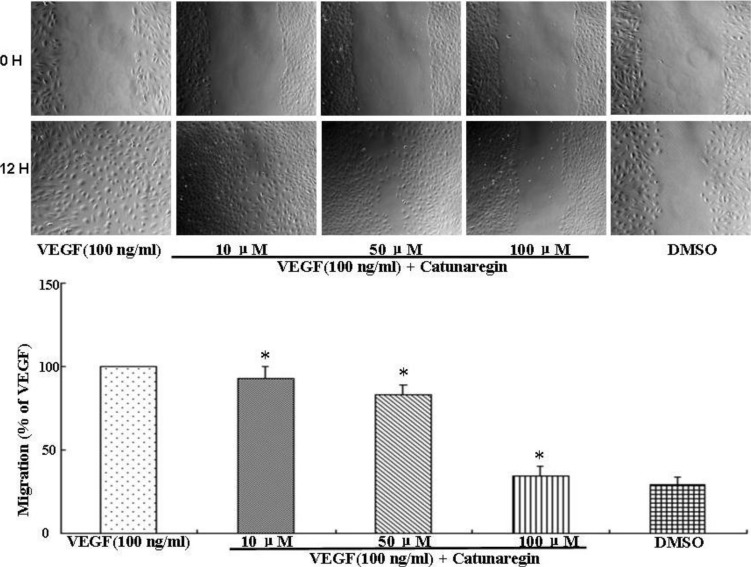
Anti-angiogenic effect of catunaregin in migration of human umbilical vein endothelial cells (HUVECs). Representative fluorescence microscopy images. The bar chart shows quantitative data for HUVECs migration with different treatments (* VEGF *vs.* catunaregin plus VEGF, *p* < 0.01).

**Figure 2:**
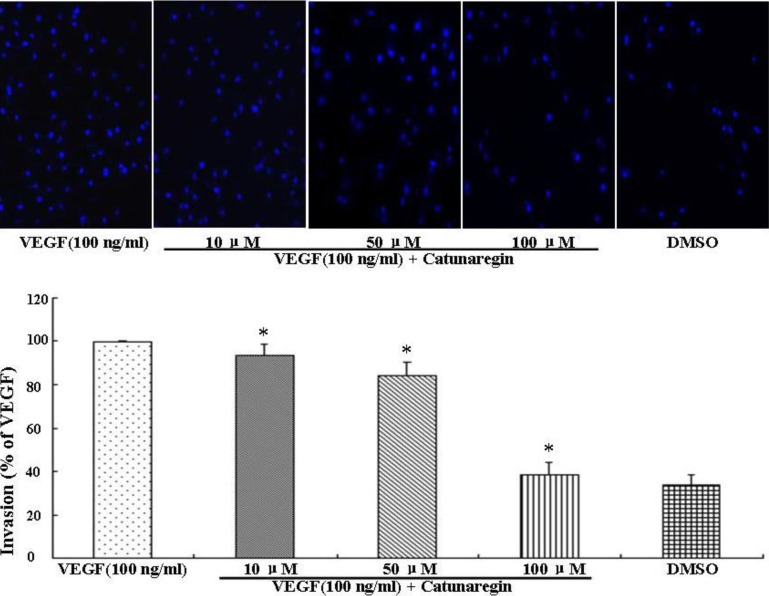
Anti-angiogenic effect of catunaregin in invasion of HUVECs. Representative fluorescence microscopy images. The bar chart shows quantitative data for HUVECs invasion with different treatments (* VEGF *vs.* catunaregin plus VEGF, *p* < 0.01).

**Figure 3:**
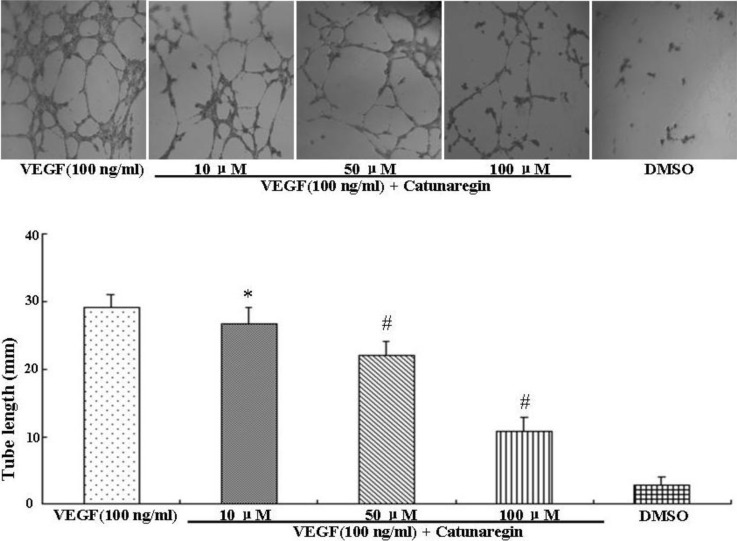
Anti-angiogenic effect of catunaregin in tube formation of HUVECs. Representative fluorescence microscopy images. The bar chart shows quantitative data for HUVECs tube formation with different treatments. (* VEGF *vs.* catunaregin plus VEGF, *p* < 0.05; ^#^ VEGF *vs.* catunaregin plus VEGF, *p* < 0.01).

Angiogenesis is the process of forming blood vessels from preexisting vessels. In response to VEGF, endothelial cells migrate into the surrounding extracellular matrix where they form blood capillaries, and thus, cell migration is a crucial step in angiogenesis and invasion. In this study, the anti-angiogenesis of catunaregin was first examined using the wound-healing method. Wound-healing assays was performed on HUVECs treated with catunaregin at concentrations of 10, 50, and 100 μM. As shown in Figure 1, treatment of HUVECs with catunaregin at these concentrations significantly prevented cell migration. The percentage decreases were 6.7%, 16.6% and 65.4% at 100 ng/mL VEGF plus catunaregin at 10, 50, and 100 μM, respectively. To further assess the anti-angiogenesis of catunaregin, a transwell invasion assay, the most popular *in vitro* test of angiogenesis [5]. During this assay, cells were seeded onto the upper surface of an 8 mm pore size membrane separating upper and lower chambers. The upper chamber contained catunaregin in 0.1% endothelial basal medium (EBM), and cellular invasion through the membrane was induced when VEGF was present in the lower chamber. The invasion assay showed that catunaregin significantly reduced VEGF-induced invasion of HUVECs. The percentage decrease were 6.4%, 16% and 61.3% at 100 ng/mL VEGF plus catunaregin at 10, 50 and 100 μM, respectively (Figure 2). The anti-angiogenesis of catunaregin was further examined using tube formation assay. In the tube formation assay, HUVECs were seeded on VEGF-reduced two-dimensional Matrigel. Consistent with previous reports, robust tubular structures were formed in the presence of VEGF whereas preincubation with catunaregin markedly and dose-dependently abolished VEGF-induced tube formation (Figure 3).

### 2.3. Catunaregin Inhibited Angiogenesis through Modulation of AKT and eNOS Signaling Pathways

**Figure 4:**
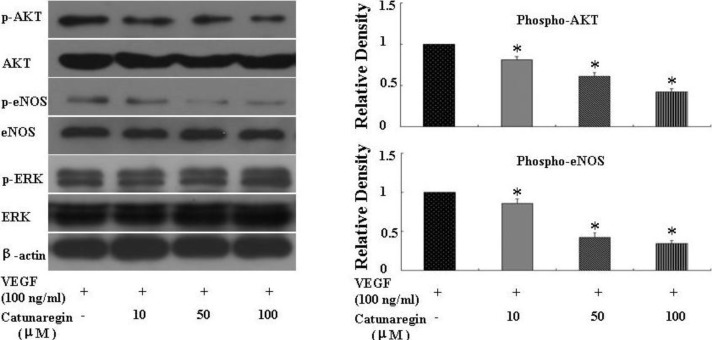
Catunaregin decreases phosphorylation of AKT and eNOS expression in HUVECs. Catunaregin significantly and concentration-dependently suppressed the VEGF-triggered phosphorylations of AKT and eNOS, whereas only mildly inhibited VEGF-induced phosphorylation of ERK 1/2 in HUVECs (* VEGF *vs.* catunaregin plus VEGF, *p* < 0.01).

Activation of Akt/eNOS and phosphorylation of ERK 1/2 are two most important mediators in VEGF-induced angiogenesis. To elucidate the mechanisms that underlie the anti-angiogenic effect of catunaregin, we examined these two signaling pathways using Western blotting. As shown in Figure 4, catunaregin significantly and concentration-dependently suppressed the VEGF-triggered phosphorylations of AKT (Ser473) and eNOS (Ser1172), whereas only mildly inhibited VEGF-induced phosphorylation of ERK 1/2 in HUVECs. Therefore, we believe that catunaregin inhibits angiogenesis possibly by modulating the AKT and eNOS signaling pathways.

### 2.4. Catunaregin Inhibited Angiogenesis in Zebrafish Embryo and Caudal Fin Regeneration Assays

**Figure 5:**
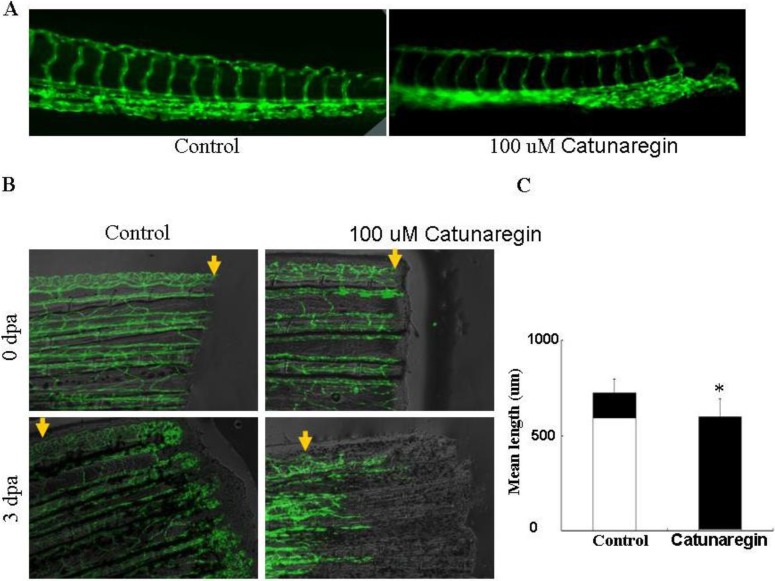
Inhibition of the zebrafish neovascularization by catunaregin. (**A**) Live fluorescent zebrafish embryo assay. Transgenic TG (fli1:EGFP) zebrafish embryos, which show green fluorescent protein (GFP) expression in endothelial cells, were incubated for 72 h without or with 100 μM catunaregin. (**B**) Caudal fin regrowth is limited by angiogenesis. The same fins are shown in bright field (top panels) and with the corresponding endothelial-eGFP signal (bottom panels). Zebrafish tail fins were clipped, then allowed to recover normally or treated with catunaregin as indicated. (**C**) Quantitative comparison of vessel and fin regeneration in control and catunaregin-treated fish. Black bars, nonvascularized fin tissue; white bars, vascularized tissue. Average values are plotted for fin and vessel growth (*n* = 15) (* catunaregin *vs.* control, *p* < 0.01).

To test whether the results obtained from *in vitro* studies is reproducible *in vivo*, we examined the anti-angiogenesis of catunaregin in zebrafish embryo and caudal fin regeneration assays in transgenic TG (fli1:EGFP) zebrafish. In these transgenic animals, enhanced EGFP are expressed in all endothelial cells which allow observation of bright blood vessels at all stages of embryogenesis. We first examined the anti-angiogenesis of catunaregin on the development of caudal intersegmental vessels at late tail bud stages of TG (fli1:EGFP) transgenic zebrafish embryos. In agreement with the results obtained from *in vitro* experiments, catunaregin significantly reduced the number of caudal intersegmental vessels in a dose-dependent manner. We further tested the specificity of anti-angiogenesis of catunaregin in zebrafish caudal fin regeneration assay because this assay can separate regenerative angiogenesis from tissue regrowth [6]. In caudal fin regeneration experiments, zebrafish caudal fins were amputated at mid-fin level, and then allowed to recover. Amputated blood vessels healed their ends by one day post amputation (dpa) and then reconnect arteries and veins via anastomosis, to resume blood flow at wound sites by 2 dpa. By 3 dpa, networks of endothelial cells in the regenerated tissue formed a vascular plexus that extended to the fin tip. When 100 μM catunaregin was added to water, new vessel formation was prevented and fin regeneration was arrested as evidenced by the absence of fin blastema. Although angiogenesis is essential for the regeneration of a complete fin, we found that limited fin tissue could still be formed in the absence of new blood vessels. Within the new formed tissue, skin and pigment cells appeared intact by visual inspection, indicating that catunaregin may specifically inhibited regenerative angiogenesis (Figure 5).

## 3. Experimental Section

### 3.1. Preparation of Catunaregin

Reagents and solvents were commercial quality and used without further purification, except for re-purified methanol used in HPLC system. NMR spectra were recorded on Bruker DRX-500 spectrometer (Bruker, Bremen, Germany) with SiMe4 (Cambridge Isctope Laboratories, Tewksbury, MA, USA) as internal standard. HRESI-MS was recorded on VG Auto Spec-3000 MS spectrometer (Bruker, Bremen, Germany). Thin layer chromatography (TLC) was carried out on precoated silica gel G plates (Qingdao Haiyang Chemical Plant, Qingdao, China) and spots were visualized by spraying the plates with 50% H_2_SO_4_ solution, followed by heating. Semi-preparative RP-HPLC was carried out on ODS columns (YMC-Pack ODS-5-A, 250 × 10 mm, 5 μm, YMC, Kyoto, Japan) with the CH_3_OH–H_2_O solvent system as eluents. Waters 600 HPLC system (Waters, Voorhees, NJ, USA) was equipped with a Waters 996 photodiode array detector (Waters, Voorhees, NJ, USA) to check the fraction and purity of target compound.

### 3.2. The Isolation of Catunaregin

Catunaregin was isolated and purified by CAS Key Laboratory of Tropical Marine Bio-resources and Ecology, South China Sea Institute of Oceanology, Chinese Academy of Sciences, Guangzhou, China. The purity of the compound was >98%. Catunaregin was dissolved in dimethyl sufoxide (DMSO) and stored at −20 °C until use. The solution form of catunaregin was then diluted by PBS to the concentration needed. All reagents were purchased from Sigma (Sigma, Shanghai, China) unless otherwise stated.

### 3.3. Preparation of Reagents and Cell Culture

Human umbilical vein endothelial cells (HUVECs) were obtained from Sciencell, Carlsbad, NM, USA. HUVECs (Passages 4 to 7) were cultured at 37 °C in M199 media (Invitrogen, Carlsbad, NM, USA) supplemented with 10% FBS. Cells were maintained in a humidified atmosphere of 5% CO_2_. The anti-angiogenic effect of catunaregin on HUVECs was evaluated using a stock solution of catunaregin (1 mg/mL) prepared in PBS containing 10% DMSO (GIBCO, Langley, VA, USA). The anti-angiogenic effect on zebrafish was evaluated using a stock solution of catunaregin (1 mg/mL) prepared in sterilized Milli-Q water containing 0.5% DMSO. Vascular endothelial growth factor (VEGF) was obtained from Sigma, St. Louis, MO, USA and prepared as a stock solution of 100 μg/mL in sterilized Milli-Q water.

### 3.4. Cell Invasion, Migration and Tube Formation

HUVEC migration assay was performed using the wound healing method as previously described [7,8]. The HUVECs (3 × 10^5^ cells) were seeded into each well of a 24-well plate and incubated with complete medium at 37 °C and 5% CO_2_. After 24 h of incubation, cells were starved for additional 24 h by low serum (0.5% FBS) medium. The HUVECs were then scraped away horizontally in each well using a P100 pipette tip (Axygen, Union city, CA, USA). Three randomly selected views along the scraped line were photographed on each well using a fluorescent inverted microscope (Olympus, Tokyo, Japan) and the CCD camera (Olympus, Tokyo, Japan) attached to the microscope at 50× magnification. The medium was then changed to fresh low serum (1% FBS) medium containing DMSO with or without vascular endothelial growth factor (VEGF; 100 ng/mL) and the indicated concentration of the compound of catunaregin. The final concentration of DMSO was 0.1% in all experimental groups. After 12 h of incubation, another set of images were taken by the same method. Image analysis for signs of migration was performed by Metamorph Imaging Series (Molecular Devices, Tokyo, Japan). The average scraped area of each well under each condition was measured and subtracted from that of the before-treatment condition. Data are expressed as percentage wound closure relative to the wound closure area in the control medium. The wound closure area of the control cells was set at 100%.

HUVEC invasion was investigated as described [5,9]. Briefly, the effect of catunaregin on HUVEC invasion was measured using the 10 mm tissue culture insert (transwell) with polycarbonate membrane (8 mm pores) and 24-well companion plate. The upper side and lower side of the membrane were pre-coated with 1:30 (v/v) and 1:100 (v/v) of Matrigel, respectively. The HUVECs were resuspended in low serum (1% FBS) medium and seeded onto the culture inserts at 5 × 10^4^ cells per insert in triplicate. They were then deposited into the 24-well companion plate with 500 μL of low serum (1% FBS) medium containing DMSO with or without vascular endothelial growth factor (VEGF; 100 ng/mL) and the indicated concentration of the compound of catunaregin. The final concentration of DMSO was 0.1% in all experimental groups. The inserts were removed after 8 h of incubation and were then washed with PBS. Non-invasive cells on the upper surface of the membrane were removed by wiping with cotton swabs. The inserts were fixed in paraformaldehyde, stained with DAPI and mounted on microscope slides. Images of the invasive cells were captured at 100× magnification using a fluorescent inverted microscope and a CCD camera. Following this, HUVEC invasion was quantified by counting the number of cells per insert with the software Metamorph Imaging Series (Molecular Devices, Tokyo, Japan).

Endothelial tube formation was assessed in 24-well plates using growth factor-reduced Matrigel™ as described previously [10,11]. Briefly, growth factor-reduced Matrigel (250 μL) was pipetted onto 24-well culture plates and polymerized for 30 min at 37 °C. HUVECs were seeded on Matrigel-coated plates at a density of 5 × 10^4^ in low serum (1% FBS) medium containing DMSO with or without vascular endothelial growth factor (VEGF; 100 ng/mL) and the indicated concentration of the compound of catunaregin. The final concentration of DMSO was 0.1% in all experimental groups, then incubated at 37 °C for 8 h. The network-like structures were examined under an inverted microscope (at 50× magnification). Tube-like structures were defined as endothelial cord formations that were connected at both ends. The tube length was quantified using the software NIH Image (NIH, Bethesda, MD, USA) as reported earlier [10,11].

### 3.5. Western Immunoblot Analysis

HUVECs were plated in 60-mm dishes (16,700 cells per centimeter square) and cultured for 2 days. The culture medium was then replaced with fresh medium containing 2% FBS, and the cells were treated with indicated concentration of the compound of catunaregin for 24 h. Whole-cell protein extracts (20 μg) were separated on 10%–12.5% sodium dodecylsulfate polyacrylamide gels, and transferred to nitrocellulose membranes (Amersham, Pittsburgh, PA, USA). The blotted membranes were incubated with antibodies against Akt, phosphorylated Akt (Ser473), endothelial nitric oxide synthase (eNOS), phosphorylated eNOS (Ser1177) and β-actin. Densitometrical results were calculated by NIH image software. The primary antibodies used were antiphospho-ERK1/2 (Cell Signaling Technology, Danvers, MA, USA), antibody to total ERK1/2 (Cell Signaling), anti-eNOS (Santa Cruz Biotechnology, Santa Cruz, CA, USA), anti-phosphorylation of eNOS at S1177 (P-eNOS S1177) (Cell Signaling), anti-phospho-Akt (Cell Signaling), anti-Akt (Cell Signaling), and anti-β-actin (Biosynthesis Biotechnology, Beijing, China).

### 3.6. Zebrafish Embryo Assay

The transgenic zebrafish cell line TG (fli1:EGFP), in which endothelial cells expressed eGFP, was kindly provided by ZFIN (Eugene, OR, USA) and maintained as described [11,12]. Zebrafish embryos were generated by natural pair-wise mating of fish that were between 3 and 12 months old. Embryos were collected as described. Healthy embryos were harvested at the 1–4 cell stage, plated in a 96-well microplate and incubated with 100 μL of the indicated concentrations of the tested compounds at 28 °C for 24 h. DMSO was used as both carrier of drugs and control. After incubation, fish embryos were anesthetized with tricaine (0.02%), placed on slides and examined under an OLYMPUS IX71 fluorescence inverted microscope (Olympus, Tokyo, Japan). Phenotypic changes were evaluated by two different observers.

### 3.7. Zebrafish Caudal Fin Regeneration Assay

Adult TG (Fli-EGFP) transgenic fishes were anesthetized with tricaine (0.02%), and their caudal fin was partially amputated. Then, fishes were maintained for 3 days at 28 °C in 100 μL water containing the indicated concentration of the compound. After three days, fishes were anesthetized and examined and photographed under an OLYMPUS IX71 fluorescence inverted microscope.

### 3.8. Statistical Analysis

All data were presented as mean ± SEM. Differences among test groups were analyzed by ANOVA, using Newman-Keuls multiple comparison test (Prism 4.0, GraphPad Software, Inc., San Diego, CA, USA). A *p* value <0.05 was considered statistically significant.

## 4. Conclusions

This is the first study concerning the anti-angiogenic action of catunaregin. Our experimental results demonstrated that the catunaregin could significantly inhibit VEGF-induced different angiogenic phenotypes of HUVECs *in vitro* and the vessel formation in transgenic zebrafish. To further investigate the underlying mechanisms behind the anti-angiogenic property of catunaregin, we examined angiogenesis-related signaling pathways in HUVECs. Our results demonstrated that catunaregin inhibited the phosphorylation of Akt and eNOS in HUVECs, indicating a new mode of biological activity for catunaregin. Interestingly, catunaregin inhibited regenerative angiogenesis without severely affecting tissue regrowth. Thus, catunaregin may primarily inhibit the formation of new blood vessels through blockade of angiogenic process of endothelial cells. Given the critical role of Akt and eNOS signaling pathways in tumorigenesis and tumor metastasis, our results provide an impetus for further investigation and development of catunaregin as a novel anti-angiogenesis candidate for the treatment of diseases with increased angiogenesis. 

Catunaregin is a novel norneolignan with a unique O-bridged furopyran ring. It can produce hemiacetal compound when 7-position is cleaved via hydrolysis. Moreover, hemiacetal compound can be open loop at 1-position and 5-position through acetal hydrolysis, thereby affording diketone intermediate. Thus, unsubstituted hydroxyl group or easily activating hydroxyl group like hemiacetal at 6-position and 8-position of compound may be important for its anti-angiogenic action.
